# Centralized and Interdisciplinary Therapy Management in the Treatment of Sarcomas

**DOI:** 10.3390/life13040979

**Published:** 2023-04-10

**Authors:** Annika Strönisch, Sven Märdian, Anne Flörcken

**Affiliations:** 1 Department of Hematology, Oncology, and Tumor Immunology, Charité-Universitätsmedizin Berlin, Corporate Member of Freie Universität Berlin and Humboldt-Universität zu Berlin, Campus Virchow-Klinikum, Augustenburger Platz 1, 13353 Berlin, Germany; 2 Centre for Musculoskeletal Surgery, Charité-Universitätsmedizin Berlin, Corporate Member of Freie Universität Berlin and Humboldt-Universität zu Berlin, Campus Virchow-Klinikum, Augustenburger Platz 1, 13353 Berlin, Germany; 3 Charité-Universitätsmedizin Berlin, German Cancer Consortium (DKTK), Partner Site Berlin, and German Cancer Research Center (DKFZ), 69120 Heidelberg, Germany

**Keywords:** sarcoma, interdisciplinary therapy, multidisciplinary therapy, interdisciplinary tumour board, cancer centre, expert centre

## Abstract

Sarcoma treatment requires a high level of expertise due to its rarity and heterogeneity. Sarcoma patients should, therefore, be referred to an expert centre as early as possible to ensure optimal treatment. Numerous studies have been carried out to provide evidence for this strategy. In compliance with the 2020 PRISMA guidelines, a systematic search was conducted in PubMed, EMBASE, Ovid Medline, ClinicalTrials.gov and Cochrane Library databases. The subject of these studies was the centralised treatment of adult sarcoma patients at expert centres and the use of interdisciplinary tumour boards. Uncertainty in therapy, delays in referral to expert centres, and limited access to therapeutic modalities continue to be a challenge in sarcoma therapy. At expert centres, diagnostic procedures were more frequently and adequately performed, and treatment was associated with an improvement in outcomes in the majority of studies: patients benefited from longer survival, lower local recurrence rates and a better postoperative outcome. The implementation of an interdisciplinary tumour board was associated with discrepant results. In a greater number of studies, it was associated with a lower local relapse rate, better overall survival and surgical outcome. In two studies, however, a shorter overall survival was observed. The establishment of expert centres and the consistent use of interdisciplinary tumour boards are important structures for ensuring multidisciplinary therapy approaches. There is growing evidence that this holds great potential for optimising sarcoma therapy.

## 1. Introduction

In rare and ultra-rare cancers, interdisciplinary and centralized management is considered mandatory for optimal treatment. Accounting for 1% of all cancers, sarcomas belong to rare tumour entities [1]. They occur comparatively frequently during childhood and young adulthood and can manifest in every region of the body as they derive from the mesenchymal tissue. The current WHO classification describes over one hundred different subtypes [1]. Due to their resulting heterogeneity, sarcomas have constantly challenged practitioners. The rather complex multimodal concepts in sarcoma therapy need careful planning. Therefore, the management of sarcomas, from diagnostics to therapy and follow-up care, should be carried out as early as possible at an expert centre [2].

Centralizing sarcoma treatment in expert centres is not a new concept. For example, in 1994, Gustafson et al. reported a lower local recurrence rate and a reduced need for re-resection in a Swedish cohort with soft tissue sarcomas if they were presented preoperatively to an expert centre [3]. The current guidelines of the European Society for Medical Oncology (ESMO) characterize expert centres as follows: in addition to a high number of cases, an expert centre should have the appropriate resources to carry out diagnostics and therapy according to the current guidelines and be involved in the latest research. Furthermore, the involvement of multiple disciplines in sarcoma treatment is a requirement, which is expressed, among other things, to establish a weekly tumour board [4].

In 1987, a self-proclaimed group of experts was built at Helsinki University Hospital, Finland. The members came from oncology, radiotherapy, orthopaedics, plastic surgery, pathology, and radiology. A treatment protocol was designed based on the current literature at that time. Before starting therapy, all new cases diagnosed with soft tissue sarcoma were discussed in weekly meetings. This approach improved disease-free survival and local control rates [5]. These interdisciplinary meetings differ only slightly from the multidisciplinary tumour boards held today. The German Cancer Society demands the presence of a specialist in radiology, pathology, haematology/oncology, surgery, and radiotherapy, as well as other disciplines, if required, on the multidisciplinary tumour board as part of the certification of sarcoma centres in Germany [6].

As soon as sarcoma is suspected, further diagnostics and treatment should be performed at an expert centre [2,4]. The guidelines specify the precise diagnostic steps for both soft tissue and bone sarcomas. At first, the primary tumour should be documented by an MRI scan. Then, a complete staging through a CT scan should be performed before starting therapy [2,4,7], followed by a tumour biopsy which should be obtained preferably by an expert centre [2]. A punch or incisional biopsy has proven to be favourable for histological diagnosis. The biopsy’s route should be chosen so it may be removed during future resection. Fine-needle biopsies are not recommended. An excisional biopsy can be evaluated for small, superficial tumours [2]. The histopathological evaluation should be conducted by a pathologist experienced in sarcoma based on the current 2020 WHO classification [2,4,7].

Before the therapy, patients should be presented to an interdisciplinary tumour board for careful and individual therapy planning. Further consultation with the multidisciplinary tumour board is also recommended following resection in the case of tumour recurrence or if the therapy regime has to be changed for any reason [2].

In a localized tumour stage, a complete resection of the tumour is warranted regardless of the sarcoma type. As a standard, wide resection is performed by removing the tumour in one part and encapsulating it with healthy tissue. The achievement of free tumour margins (R0 status) is from prognostic significance. If an R0-status is not attained, patients should be referred to an expert centre to evaluate a reresection [2,4,7].

Prognostic factors such as the tumour characteristic, localisation and diameter, and clinical presentation must be assessed thoroughly to evaluate the necessity of a multimodal therapy concept [2,4]. Depending on this risk assessment, radiotherapy and chemotherapy were used in a neoadjuvant or adjuvant setting on the condition that the present sarcoma type was sensitive to the considered therapy modality.

Anthracyclines are the most frequently used chemotherapeutic substances in the treatment of sarcomas, either as a monotherapy or in combination with other substances [2,4,7]. In addition, the exclusive treatment of soft-tissue sarcoma techniques as regional deep-wave hyperthermia in combination with either chemo- or radiotherapy and isolated-limb perfusion is established and supplements the multimodal concept in individual cases [2].

In a metastasized tumour stage, systemic therapy builds the foundation of the treatment. The resection of metastasis should be decided individually and can improve survival. Radiotherapy and local ablative treatment can also be evaluated if resection of the metastasis is not possible [2,4,7].

Centralisation and interdisciplinary approaches to therapy are of particular significance in treating sarcomas. There is a consensus that patients can benefit from this strategy. Multiple works and studies were performed to establish evidence for this therapeutic principle and characterize the extent to which this principle is implemented in everyday clinical practice. This review aims to systematically summarize the available literature results and create an overview of their statements.

## 2. Materials and Methods

We performed a systematic review that was concordant with the updated guidelines of the 2020 Preferred Reporting Items for Systematic Reviews and Meta-Analyses for non-randomised studies (PRISMA; Supplementary Material File S1: The PRISMA 2020 study protocol checklist) [8].

PubMed, EMBASE, Ovid Medline, ClinicalTrials.gov, and Cochrane Library were systematically searched for eligible studies. The following MeSH terms were used for the databases PubMed, EMBASE, and Ovid Medline:

(“sarcomas” OR “sarcoma”) AND (“specialist centre” OR “reference centre” OR “expert centre” OR “expert network” OR “tumour conference” OR “tumor conference” OR “tumour conferences” OR “tumor conferences” OR “tumour board” OR “tumor board” OR “tumor boards” OR “tumour boards” OR “interdisciplinary tumor board” OR “interdisciplinary tumor board” OR “multidisciplinary tumour board” OR “specialized centre” OR “sarcoma centres” OR “multidisciplinary management” OR “interdisciplinary management” OR “multidisciplinary treatment” OR “interdisciplinary treatment” OR “multidisciplinary therapy” OR “interdisciplinary therapy”).

The registers ClinicalTrials.gov and Cochrane Library were searched by using the term “sarcoma” combined with one of every item used in the MeSH terms mentioned above. The last search was conducted on 12 July 2022 without the application of any limitations. All English and German studies published until 12 July 2022 were considered.

The search results were evaluated by the first reviewer (A.S.). Articles that did not correspond to the searched study type, duplicates, incomplete studies, conference abstracts, and studies incoherent to the inclusion and exclusion criteria were excluded (Table 1).

All primary mono- and multicentric studies addressing the centralized treatment of adult sarcoma patients at expert centres and the involvement of an interdisciplinary tumour board were included. This comprised retro- and prospective studies, with data obtained from cancer registries, databases, structured qualitative interviews, and cross-sectional surveys. Reviews as well as meta-analyses were excluded as the focus of this paper was on primary studies. The review was registered on OSF (www.osf.io).

### 2.1. Quality Control and Assessment

Both the abstracts and full texts of eligible studies were methodically analysed by the first reviewer (A.S.). The included studies were separated into groups (I and II) for practical reasons regarding the risk of bias assessment, even though the studies overlapped thematically (Table 2 and Table 3). All studies conducted an outcome analysis of interdisciplinary and centralized sarcoma treatment and, therefore, were comparable to a randomised trial and assigned to group I (named ‘patient outcome analysis’). The remaining studies, including interviews and cross-sectional surveys of an entirely descriptive nature, were allocated to group II (named ‘status of sarcoma care’).

In group I, a risk of bias assessment was performed for every study using the Risk of Bias In Non-randomized Studies—of Interventions (ROBINS-I) tool [9]. The Cochrane Bias Methods Group built the ROBINS-I tool, which contains seven bias domains to evaluate potential bias compared to an ideal, ‘theoretical’ randomized trial on the effect of an intervention [9] (Table 4).

Each study of group II was individually examined retrospectively for possible limitations and risk of bias. In addition, the reviewers observed the limitations discussed by the study authors (Table 5). The ROBINS-I tool was not applicable since it was not feasible to define an intervention for the studies included in group II.

Following the initial evaluation by the first reviewer (A.S.), every study was carefully reassessed by the second reviewer (A.F.) Any discrepancies in the assessment were thoroughly discussed and eventually resolved.

**Table 2:** ‘Group I—patient outcome analysis’: The table summarizes the study and patient’s characteristics, results on the treatment site and the interdisciplinary tumour board and other relevant results. Outcome analysis is reported in three sections for overall survival, recurrence and surgical outcome. Abbreviations: DSS (disease-specific survival), DFS (disease-free survival), E (expert), EC (expert centre), HR (hazard ratio), ITB (interdisciplinary tumour board), LR (local recurrence), LRFS (local recurrence-free survival), MD (metastatic disease), N/A (not available), NEC (non-expert centre), OS (overall survival), PFS (progression-free survival), OR (odds ratio), RFS (recurrence-free survival), S (surgeon).

Author	Year	Type of Trial	n	Median Age (Years)	Sex Ratio (Female:Male in %)	Sarcoma Type	Treatment Site	Interdisciplinary Tumour Board	Treatment and Other	Median Follow Up (Range)	Overall Survival	Recurrence	Surgical Outcome
Blay et al. [10]	2017	Retrospective data base study, multicentric, prospectively obtained data	12,528 (9646 for survival analysis)	18–101 (61)	51.1:49.6	Soft tissue sarcoma	N/A	Primary tumour imaging (*p* < 0.001) with tumour board 87.9% w/o tumour board 60.4% Diagnostic biopsy (*p* < 0.001) with tumour board 87.7% w/o tumour board 41.9%	N/A	26 months	N/A	Treatment before tumour board presentation 2 year-LRFS (*p* < 0.001) before 65.4% after 76.9% 2 year-RFS (*p* < 0.001) before 46.6% after 51.7% multivariate analysis LRFS Treatment before tumour board HR 1.8 (*p* < 0.001) RFS Treatment before tumour board HR 1.3 (*p* < 0.001)	R0-status at primary surgery (*p* < 0.001) with tumour board 52.6% w/o tumour board 32.2% Re-excision (*p* < 0.001) with tumour board 6.0% w/o tumour board 17.4%
Jagodzińska-Mucha et al. [11]	2020	Retrospective cohort study, monocentric	180	26 (18–67)	39:61	Ewing-sarcoma	Initial treatment at EC 72% NEC 28%	N/A	N/A	38.5 (8.3–160) months	Median OS 52 (24–79) months Admission after biopsy to EC within 3 months, 5 years OS 50% >3 months, 5 years OS 50% multivariate analysis treatment in <3 months from time of biopsy HR 1.6 (95%-CI 0.97–2.76)	Median PFS 26 (20–32) months PFS 5years for admission after biopsy to EC (*p* < 0.001) within 3 months 28% >3 months 14%	N/A
Gilg et al. [12]	2020	Retrospective cohort study, monocentric	109	66 (21–96)	51:49	Myxofibrosarcoma	Primary Surgery at EC 62% NEC 38%	N/A	N/A	42 (2–273) months	OS 3 years 80% OS 5 years 76%	LRFS 3 years 95% LRFS 5 years 88% OR 8.5 (95%-CI 1.59–49.79) (*p* = 0.01) for R1/2-status at primary surgery	R0-status at primary surgery (*p* < 0.001) EC n = 68 R0 85.3% R1/2 14.7% NEC n = 41 R0 12.2% R1/2 87.8%
Venigalla et al. [13]	2018	Retrospective observational cohort study, multicentric, prospectively obtained data	9205	61 (49–73)	44:56	Soft tissue sarcoma	Treatment at EC 17% NEC 83%	N/A	Chemotherapy EC 23% NEC 13% Neoadjuvant radiotherapy EC 37% NEC 19% Adjuvant radiotherapy EC 63% NEC 81%	47.9 (2.3–142.6) months	OS 5 years (*p* < 0.001) EC 72.2% NEC 57.1% Median OS (*p* < 0.001) EC 11.6 years NEC 9.8 years multivariate analysis EC HR 0.81 (95%-CI 0.72–0.90) NEC reference	N/A	Positive surgical margins (*p* < 0.001) EC OR 0.72 (95%-CI 0.60–0.87) NEC Reference
Marec-Bérard et al. [14]	2020	Retrospective data base analysis, multicentric, prospectively obtained data	140	Age 13–17 n = 71 Age 18–25 n = 69	37:63	Sarcomas in general	EC University hospital 30% Cancer centre 53% NEC 17%	At least one tumour board 85%	N/A	11.44 (95%-CI 9.95–11.75) years	OS 5 years 62% (95%-CI 53–70) OS 10 years 57% (95%-CI 48–65) median OS 14.24 years multivariate analysis Discussion in ITB (*p* = 0.49) HR 1.39 (95%-CI 0.54–3.57)	RFS 5 years 59% (95%-CI 50–66%), RFS 10 years 48% (95%-CI 39–57%) median RFS 9.13 years	N/A
Snow et al. [15]	2018	Retrospective data base analysis, monocentric, prospectively obtained data	88	EC 59.5 (18–86); NEC 56 (34–73)	EC 31:69 NEC 58:42	Retroperitoneal soft tissue sarcoma	Primary Surgery at EC 70.5% NEC 29.5% Diagnostic core biopsy EC 92% NEC 31% No/surgical biopsy/partial rection (*p* < 0.001) EC 8% NEC 69%	N/A	Neoadjuvant radiotherapy EC 87% NEC 12% Adjuvant radiotherapy EC 2% NEC 19% Palliative radiotherapy EC 0% NEC 8%	36 months	OS 5 years 66% (95%-CI 52–83%) Univariate analysis—OS Biopsy technique (*p* = 0.55) No/surgical biopsy/partial resection. HR 1.4 (95%-CI 0.51–3.6) Diagnostic core biopsy reference Primary surgery (*p* > 0.99) EC HR 1.0 (95%-CI 0.37–2.7) NEC reference Neoadjuvant radiotherapy (*p* = 0.93) No HR 1.0 (95%-CI 0.4–2.7) Yes reference	LRFS 5 years 65% (95%-CI 52–80%) Univariate analysis—LR Biopsy technique (*p* = 0.019) No/surgical biopsy/partial resection HR 2.8 (95%-CI 1.1–7.0) Diagnostic core biopsy reference Primary surgery (*p* = 0.055) NEC HR 2.4 (95%-CI 0.96–5.8) EC reference Neoadjuvant radiotherapy (*p* = 0.014) Yes HR 0.33 (95%-CI 0.13–0.84) No reference	Macroscopically complete resection (*p* < 0.15) EC 97% NEC 88%
Kreyer et al. [16]	2018	Retrospective data base analysis, monocentric, prospectively obtained data	481	16.5 (0.6–69.3)	39.5:60.5	Ewing-sarcoma	N/A	Recommendation Received 36.2 Not received 63.8% Compliance to recommendations 77%	Combined local treatment in localized disease. Surgery and Radiotherapy recommendation (*p* = 0.021) Received 49.1% Not received 35.9%	3.2 (0.2–7.6) years	Lower OS for non-compliance to tumour board recommendation compared to compliance and recommendation or no recommendation. Better OS for metastatic disease with compliance to recommendations than w/o recommendation (*p* = 0.028) Similar outcome in localized disease with or w/o recommendation	N/A	Radical/wide surgical margins in localized disease (*p* = 0.325) Recommendation Received 56.1% Not received 56.7%
Bonvalot et al. [17]	2019	Retrospective data base analysis, monocentric, prospectively obtained data	2945	60.8 (15–94)	49.4:50.6	Retroperitoneal soft tissue sarcoma	Primary Surgery at EC 36.6% NEC 63.4% Adequate imaging at (*p* < 0.001) EC 88.7% NEC 71.8% Biopsy at (*p* < 0.001) EC 79% NEC 54.2%	Tumour board before surgery at (<0.001) EC 51.2% NEC 29.8%	Adjuvant treatment EC 10.3% NEC 5%	22 months	OS 2 years for treatment at (*p* < 0.001) EC 87% NEC 70% Multivariate analysis Primary surgery at (*p* < 0.001) EC OR 0.496 NEC reference Tumour board presentation was not a prognostic factor	LRFS 2 years (*p* < 0.001) EC 75% NEC 55% Multivariate analysis LRFS Primary surgery at (*p* < 0.001) EC OR 0.530 NEC reference Multivariate analysis PFS Primary surgery at (*p* < 0.001) EC OR 0.604 NEC reference	R-Status at primary surgery R0 EC 41.9% NEC 12.5% R1 EC 33.9% NEC 17.8% R2 EC 4.5% NEC 9.2% Unknown/Not evaluable EC 19.7% NEC 60.7%
Bhangu et al. [18]	2004	Retrospective data base analysis, multicentric	260 (231 for survival analysis)	61	57.3:42.7	Soft tissue sarcoma	Initial treatment at EC 37% NEC 63%	N/A	N/A	Minimum 3year follow-up	OS 5 years 58% Multivariate analysis Initial treatment at (*p* = 0.048) EC reference NEC HR 1.7 (CI 1.01–2.8)	LR (*p* < 0.01) EC 19% NEC 39% LR for adequate margins (*p* = 0.025) EC 12% NEC 39%	Adequate margins EC 39% NEC 35%
Martin-Broto et al. [19]	2019	Prospective cohort study, multicentric	622 (high-risk 138)	55	49:51	Soft tissue sarcoma, local	Treatment at EC 54% NEC 46%	N/A	High-risk sarcoma Perioperative chemotherapy (*p* = 0.01) EC 67% NEC 45%	40 (1–97) months	OS 3 years for treatment at (*p* = 0.003) EC 82% (95%-CI 74–90) NEC 70.4% (95%-CI 64.7–76) Median OS for treatment at (*p* = 0.036) EC 30.4 (95%-CI 22.5–38.3) months NEC 18.5 (95%-CI 13.3–23.6) months	LR EC 60% NEC 44% MD (*p* = 0.058) EC 24.7% NEC 40% Coincident MD and LR was similar in EC and NEC RFS 3 years after biopsy at (*p* = 0.019) EC 66% (95%-CI 56.1–75.9) NEC 46.4% (95%-CI 31.9–60.9) High-risk sarcoma RFS 3 years for perioperative chemotherapy (*p* = 0.011) Yes 66% (95%-CI 60–72) No 44% (95%-CI 36–52) Multivariate analysis Perioperative chemotherapy (*p* = 0.013) Yes reference No HR 2.06 (95%-CI 1.16–3.64)	Positive postsurgical margins after biopsy at (*p* = 0.002) EC 21.3% NEC 42.4%
Sandrucci et al. [20]	2018	Retrospective cohort study, multicentric	72 for survival analysis	<60 years 21% >60 years 79%	40.3:59.7	Retroperitoneal soft tissue sarcoma	Treatment at EC 34.7% NEC 65.3% Biopsy at (*p* = 0.45) EC 60.0% NEC 66.5%	N/A	N/A	85 (72–100) months	OS 5 years R-Status (*p* < 0.001) R0-R1 65% R2 31%	N/A	R-Status (*p* = 0.013) EC R0/R1 80% R2 12% NEC R0/R1 49% R2 32% Resection specimen (*p* = 0.01) EC Intact 76% Fragmented 24% NEC Intact 36.2% Fragmented 63.8%
Ray-Coquard et al. [21]	2004	Retrospective cohort study, multicentric	100	58 (18–88)	56:44	Soft tissue sarcoma, local	Primary surgery at EC University hospital 28% Cancer centre 18% NEC Private institution 39% General hospital 15%	Tumour board before surgery 39% Tumour board after surgery 73%	Adjuvant therapy n = 62 Radiotherapy 29% Chemotherapy 32% Radio- and chemotherapy 39%	N/A	N/A	Median LRFS 48 (24–72) months Univariate analysis for LR Tumour board before surgery (*p* = 0.02) Yes 23% No 44% R-Status (*p* = 0.01) R0 20% R1 26% R2 51% Radiotherapy conforms to guidelines (*p* = 0.007) Yes 30% No 63% Chemotherapy (*p* = 0.08) Yes 41% No 27%	Proportion of tumour board before surgery depending on R-status (*p* = 0.001) R0 60% R1 48% R2 16% Rate of R2-resection at (*p* = 0.021) EC 27% NEC 61%
Keung et al. [22]	2018	Retrospective cohort study, multicentric, prospectively obtained data	6950 (4969 for survival analysis)	62 (18–90)	53.7:46.3	Retroperitoneal soft tissue sarcoma, local	Primary surgery at EC 9.8% NEV 90.2% Time between diagnosis and initial treatment (*p* < 0.001) EC median 28 days NEC median 5 days	N/A	Radiotherapy EC 17.2% NEC 27.9% Chemotherapy EC 15.4% NEC 13.6%	N/A	OS 5 years EC 57.7% NEC 52.0% Median OS for treatment at EC 76.2 months NEC 64.2 months Multivariate analysis Primary surgery at (*p* = 0.003) EC reference NEC HR 1.30 (95%-CI 1.10–1.55) Radiotherapy (*p* < 0.001) Yes HR 0.80 (95%-CI 0.73–0.88	N/A	R2-Status (*p* < 0.001) EC 1.6% NEC 4.5% 30-day readmission rate (*p* < 0.001) EC 1.8% NEC 3.4% 30-day mortality after surgery (*p* = 0.004) EC 1.9% NEC 3.1%
Alvarez et al. [23]	2021	Retrospective database analysis, multicentric	Ewing-sarcoma 531 Osteosarcoma 959	Ewing-sarcoma EC < 18 years 64.5% 19–39 years 28.7% > 40 years 6.7% NEC < 18 years 24.0% 19–39 years 46.3% > 40 years 29.7% Osteosarcoma EC < 18 years 60.2% 19–39 years 25.4% > 40 years 14.4% NEC < 18 years 19.0% 19–39 years 29.9% > 40 years 51.1%	Ewing-sarcoma at EC 39.6:60.4 NEC 43.4:56.6 Osteosarcoma at EC 44.0:56.0 NEC 46.5:53.5	Ewing- and osteosarcoma, local and metastasized	Ewing-sarcoma Treatment at EC 67% Osteosarcoma Treatment at EC 61.6%	N/A	Ewing-sarcoma Radiotherapy (*p* = 0.87) EC 50.8% NEC 49.7% Chemotherapy (*p* = 0.055) EC 95.2% NEC 89.7% Osteosarcoma Chemotherapy (*p* < 0.001) EC 88.2% NEC 67.9%	N/A	Ewing-sarcoma OS for treatment at EC HR 0.49 (95%-CI 0.37–0.67) NEC reference DSS for treatment at EC HR 0.49 (95%-CI 0.35–0.68) NEC reference Osteosarcoma OS for treatment at EC HR 0.78 (95%-CI 0.63–0.97) NEC reference DSS for treatment at EC HR 0.80 (95%-CI 0.62–1.02) NEC reference	N/A	N/A
Ipach et al. [24]	2011	Retrospective cohort study, monocentric	11	47.9 (25–75)	72.7:23.3	Clear-cell sarcoma	Primary surgery at EC 54.5% NEC 45.6%	N/A	N/A	Minimum 1year follow-up	OS 5years 19% Median time from diagnosis to death 36 (5–127) month Treatment at EC median time to death 58.4 (5–127) month NEC median time to death 26.8 (13–41) month	Time to MD EC 18 (9–27) month NEC 9 (4–15) month	R-Status after primary surgery at EC R0 100% NEC R0 40% R1 20% R2 40%
Abellan et al. [25]	2009	Retrospective cohort study, monocentric	174	43.7 (SD 18.8)	N/A	Soft tissue sarcoma, local	Initial Treatment Group A—directly referred to EC 57% Group B—referred to EC after primary surgery 22% Group C—referred to EC after local recurrence 21%	N/A	N/A	91.95 (SD 80.16) months, minimum 2 years follow-up	Mean OS 69.6% No sig. difference for OS in between A, B and C Multivariate analysis Between A and B: only tumour size influenced OS and DFS (*p* = 0.024)	LR (A + B vs. C *p* < 0.001) Total 21% Group A 10% Group B 13% Group C 59% DFS (*p* < 0.001) Group A 73% Group B 76% Group C 28% MD (A + B vs. C *p* < 0.001) Total 27% Group A 22% Group B 16% Group C 51%	Disregard of oncologic surgery rules Group B 39% Group C 30%
Melo Mateus et al. [26]	2020	Retrospective data base study, multicentric	1962	N/A	46:54	Sarcomas in general	Treatment at EC Never 53% Later referral 4% Always 43%	N/A	N/A	9 days	Multivariate analysis OS for Treatment at EC (*p* < 0.05) Always HR 0.60 (95%-CI 0.46–0.79) Never reference	N/A	N/A
Sobiborowicz et al. [27]	2021	Retrospective cohort study, monocentric	27	45 (21–67)	67:33	PEComa, local	Primary surgery at EC 48.1% NEC 51.9%	N/A	Neoadjuvant radiotherapy 18.5%	68.5 (95%-CI 39–101) months	OS 5years 88% (95%-CI 74–100)	Progression at time of analysis 40.7% LRFS 1year (*p* = 0.031) EC no LR at time of analysis NEC 64% (95%-CI 41–99) DFS 5years (*p* < 0.001) EC all disease free NEC 14% (95%-CI 2–80)	R-Status at EC R0 84.6% R1 15.4 NEC R0 28.6% R1 50.0% R2 7.1% RX 14.3%
Blay et al. [28]	2019	Retrospective data base study, multicentric, prospectively obtained data	35,784 (25,851 for survival analysis)	60.8 (0–106)	50.9:49.1	Sarcomas in general, local and metastasized	Primary surgery at EC 33.7% NEC 54.8% No surgery 12.4%	Tumour board before treatment 49.2%	N/A	17 months	Multivariate analysis for OS Tumour board before treatment (*p* < 0.001) HR 1.56 (95%-CI 1.42–1.72) Primary surgery at EC (*p* < 0.001) HR 0.68 (95%-CI 0.62–0.75) Neoadjuvant radiotherapy (*p* = 0.002) HR 1.34 (95%-CI 1.11–1.60)	Multivariate analysis for LRFS Tumour board before treatment (*p* < 0.001) HR 0.67 (95%-CI 0.62–0.72) Primary surgery at EC (*p* < 0.001) HR 0.65 (95%-CI 0.61–0.70) Multivariate analysis for DFS Tumour board before treatment (*p* < 0.001) HR 0.80 (95%-CI 0.76–0.84) Primary surgery at EC (*p* < 0.001) HR 0.84 (95%-CI 0.80–0.89)	R-Status (*p* < 0.001) EC R0 53.0% R1 24.0% R2 4.2% Unknown 18.8% NEC R0 19.6% R1 20.2% R2 8.5% Unknown 50.0% Reoperation (*p* < 0.001) EC 6.2% NEC 15.7%
Pollock et al. [29]	2004	Prospective cohort study, monocentric	142 (77 malignant)	40 (6–88)	N/A	Musculoskeletal tumours in general	First Diagnostic biopsy through E 79.6% S 20.4%	N/A	Adequate diagnostic material (*p* < 0.001) E 97% S 72% Suboptimal biopsy site (*p* < 0.001) E 1.8% S 38% Malignant Adjuvant radiotherapy (*p* < 0.05) E 5.3% S 20%	N/A	N/A	N/A	Malignant re-resection after biopsy through (*p* < 0.001) E 3.5% S 40% Amputation after biopsy through (*p* < 0.03) E 7% S 25%

**Table 3:** ‘Group II—status of sarcoma care’: The table summarizes studies and patient’s characteristic as well as results for management of the diagnostic approach, access to an expert centre and an interdisciplinary tumour board. Other results valuable for the review were reported in a separate section. Abbreviations: E (expert), EC (expert centre), HR (hazard ratio), ITB (interdisciplinary tumour board), N/A (not available), NEC (non-expert centre), OR (odds ratio).

Author	Year	Type of Trial	n	Median Age (Years)	Sex Ratio (Female:Male in %)	Sarcoma Type	Diagnostic Approach	Access to an Expert Centre and an Interdisciplinary Tumour Board	Other Results
Hollunder et al. [30]	2018	Retrospective data analysis, monocentric	232 patients 320 tumour board presentations	N/A	N/A	Sarcomas and muscolosceletal tumours	N/A	Compliance to therapy recommendation of the tumour board Completely implemented 59.1% Partly implemented 14.4% No assessment possible 17.8% Deviance from tumour board recommendation 8.8% Causes for deviance Patient wish 14.3% Doctor decision 25% Death before therapy 17.9% Deviant therapy ex domo 14.9% Not documented 28.6%	N/A
Goedhart et al. [31]	2020	Cross-sectional survey	25 participants (health professionals from 17 European countries)	N/A	N/A	Bone sarcomas	N/A	Depending on the country 5–95% of the patients were referred to a bone sarcoma centre. Between 20 and 98% were treated at a bone sarcoma centre. In total, 47.1% of participating countries had access to a national bone tumour board In total, 100% of participating EC had a mandatory tumour board before treatment start In total, 84% held a tumour board once a week Each tumour board discussed a median of 15 cases (range 4–40)	N/A
Eichler et al. [32]	2021	Prospective cohort study, multicentric	1309	54 (42–64)	47.7:52.2	Soft tissue sarcomas, bone sarcomas, GIST	N/A	In total, 100% of participating study centres had access to an interdisciplinary tumour board In total, 44% had access to a specialized sarcoma board Discussion of patients in a tumour board: At some stage of their disease 88.3% Before treatment started 56.1% After therapy 78% After diagnosis of metastases 85.9% *Multivariate analysis* Tumour board discussion (*p* < 0.01) Treatment at EC OR 5.39 (95%-CI 3.28–8.85) Treatment at NEC reference	N/A
Blank et al. [33]	2020	Retrospective cohort study, monocentric, prospectively obtained data	35	N/A	N/A	Sarcomas in general	Imaging of primary tumour site 100% Biopsy before primary surgery 97%	Reviewed by tumour board Imaging 97% Pathologic results 94% TNM stage 100% Plan of care 100%	N/A
Eichler et al. [34]	2019	Cross-sectional survey	214 participants (physicians)	N/A	N/A	Sarcomas in general	N/A	In total, 52.8% of practices cooperated with an EC In total, 45.7% of hospital of maximum were an EC itself Presentation to a tumour board (*p* < 0.05) Hospital of maximum care 98.7% Practice 89% Access to a specialized sarcoma tumour board Hospital of maximum care 93% Practice 22.2%	Treatment certainty: Generally uncertain 3.3% Often uncertain 7.9% Sometimes uncertain 18.7% Mostly certain 43.9% Generally certain 24.3% Report of access problems for: Regional deep-wave hypothermia 33.2% Isolated limp perfusion 39.3% FDG-PET diagnostic 27.1%
Cho et al. [35]	2019	Cross-sectional survey	58 participants (radiation oncologists)	N/A	N/A	Soft tissue sarcoma	N/A	In total, 96.6% had access to a specialised sarcoma tumour board In total, 85.5% discussed every case In total, 14.6% discussed only complicated cases	N/A
Fayet et al. [36]	2021	Retrospective cohort study, multicentric	20,101 (488)	N/A	N/A	Sarcomas in general	N/A	Access to a specialized diagnosis in an expert centre patients in mainland France 57.9% patients in overseas territories 40.8% Access to a specialized tumour board before primary surgery patients in mainland France 30.8% patients in overseas territories 25%	N/A
Weaver et al. [37]	2020	Qualitative interviews	22 patients 17 relatives 21 health professionals	patients 43 (15–78) relatives 51 (22–66) health professionals 44 (31–62)	patients 59.1:40.9 relatives 70.6:29.4 health professionals 52.4:47.6	Sarcomas in general	N/A	N/A	Identification of four main problems in sarcoma diagnostic and care: Patients’ perception of symptoms Lack of experience Difficulties of diagnosis Availability of health services
Gantzer et al. [38]	2019	Retrospective data base study, multicentric, prospectively obtained data	643	58.6 (16–93)	56.3:43.7	Soft tissue sarcoma	Imaging according to clinical practice guidelines EC 83.8% NEC 78.3% Biopsy according to clinical practice guidelines EC 59% NEC 21.5% *Multivariate analysis for* Nonconformity with guidelines for imaging (*p* = 0.001) EC OR 1 NEC OR 4.89 (95%-CI 3.12–7.84) Nonconformity with guidelines for biopsy (*p* < 0.001) EC OR 1 NEC OR (95%-CI 3.62 (1.15–7.52) Nonconformity with guidelines for pathological assessment (*p* < 0.001) EC OR 1 NEC OR (95%-CI 1.47 (1.01–2.13)	Discussion in tumour board before surgery EC 34.6% NEC 19.4%	R0-Status after primary surgery EC 48.6% NEC 20.3%
George et al. [39]	2012	Retrospective interviews and data analysis, monocentric	Soft tissue sarcoma 66 Bone sarcoma 41	Soft tissue sarcoma 63 (16–88) Bone sarcoma 49 (17–86)	38:62	Soft tissue and bone sarcoma	Median of 3 visits of healthcare professional before diagnosis	In total, 32% referred after inappropriate biopsy/excision Soft tissue sarcoma Median patient delay in reaching expert care after 1 month (0 days–10 years) Direct referral 4% Bone sarcoma Median patient delay in reaching expert care after 1.5 months (0 days–60 years) Direct referral 10%	N/A
Johnson et al. [40]	2008	Retrospective interviews and data analysis, monocentric	162	53 (16–88)	50.7:49.3	Soft tissue sarcoma	N/A	N/A	Median referral time to EC from onset of symptoms 40.4 weeks Referral to EC within 3 month 14.6% Referral to EC after more than 1 year 44.9% Median referral time from first presentation to medical professional and EC 25.0 weeks Referral to EC within 1 month 11.3% Referral to EC within 3 months 32.7% Referral to EC after more than 1 year 32.7%
Pohlig et al. [41]	2013	Cross sectional survey	25 participants (orthopaedist)	N/A	N/A	Soft tissue and bone sarcoma	Form of biopsy Open biopsies performed by 100% Punch biopsies performed by 72% Fine needle biopsy by 8% Access way for biopsy determined by Interdisciplinary cooperation 56% Interdisciplinary tumour board 20%	Discussion of pathologic results by Interdisciplinary tumour board 88% Informal interdisciplinary meeting 12%	N/A
Styring et al. [42]	2012	Retrospective cohort analysis	Cohort 1 100 (97 referred) Cohort 2 464	Cohort 1 74 (27–97) Cohort 2 56 (18–98)	Cohort 1 37:63 Cohort 2 52:48	Cohort 1 Soft tissue sarcoma Cohort 2 16% Sarcomas in general	Cohort 1 Referral before primary surgery 86 patients Imaging before referral 74 patients Fine needle biopsy before referral 38 patients	N/A	Median referral time to EC 50 days
Thway et al. [43]	2009	Retrospective cohort analysis, monocentric	277 patients 349 specimen	N/A	N/A	Soft tissue sarcoma and GIST	Discrepancies in pathological diagnosis between EC and referring to NEC Diagnostic agreement 73.4% Discrepancies 26.6% Minor discrepancies 15.7% Major discrepancies leading to changes in therapy management 10.9%	N/A	N/A
Thway et al. [44]	2014	Retrospective cohort analysis, monocentric	288 patients 350 specimens	57.5 (2–96)	61.2:38.8	Soft tissue sarcoma and GIST	Discrepancies in pathological diagnosis between EC and referring to NEC Diagnostic agreement 57.8% Minimal discrepancies 14.0% Minor discrepancies 11.8% Major discrepancies leading to change in therapy management 16.4%	N/A	N/A
Wellauer et al. [45]	2022	Retrospective cohort analysis	347 Cohort A 196 Cohort B 149	55 (12–90)	51.6:48.4	Tumours and sarcomas of the soft tissue	Discrepancies in pathological diagnosis between local and expert pathologist (Cohort A included) Diagnostic agreement 77.6% Minor discrepancies 10.2% Major discrepancies leading to changes in therapy management 12.2% Time to pathological diagnosis (*p* < 0.01) Cohort A: Specimen analysed by local and expert pathologist for 4.7 days Cohort B: Directly analysed by expert pathologist 3.3 days	N/A	N/A

**Table 4:** ‘Group I—patient outcome analysis’: Risk of bias assessment according to ROBINS-I tool [9].

Reference	Confounding Bias	Selection Bias	Classification of Intervention Bias	Deviation from Intended Intervention Bias	Missing Data Bias	Measurement of Outcome Bias	Selection of Reported Results Bias	Overall Risk of Bias
Blay et al. [10]	moderate	moderate	moderate	low *	low	moderate	moderate	moderate
Jagodzińska-Mucha et al. [11]	moderate	serious	serious	low *	low	serious	serious	serious
Gilg et al. [12]	moderate	moderate	serious	moderate *	moderate	moderate	moderate	serious
Venigalla et al. [13]	moderate	moderate	serious	moderate *	moderate	moderate	moderate	serious
Marec-Bérard et al. [14]	moderate	moderate	serious	low *	moderate	moderate	moderate	serious
Snow et al. [15]	moderate	serious	serious	low *	low	moderate	moderate	serious
Kreyer et al. [16]	moderate	moderate	moderate	serious *	moderate	moderate	serious	serious
Bonvalot et al. [17]	moderate	low	moderate	low *	low	serious	moderate	serious
Bhangu et al. [18]	serious	low	moderate	low *	serious	serious	serious	serious
Martin-Broto et al. [19]	moderate	low	moderate	low **	serious	moderate	moderate	serious
Sandrucci et al. [20]	serious	moderate	moderate	low *	low	serious	moderate	serious
Ray-Coquard et al. [21]	serious	serious	moderate	low *	moderate	serious	moderate	serious
Keung et al. [22]	moderate	low	moderate	low *	moderate	serious	serious	serious
Alvarez et al. [23]	moderate	low	moderate	low *	moderate	moderate	serious	serious
Ipach et al. [24]	critical	low	moderate	low *	low	moderate	low	critical
Abellan et al. [25]	serious	serious	serious	low *	low	serious	moderate	serious
Melo Mateus et al. [26]	serious	low	moderate	low *	low	serious	moderate	serious
Sobiborowicz et al. [27]	serious	serious	moderate	moderate *	moderate	moderate	serious	serious
Blay et al. [28]	moderate	low	moderate	low *	moderate	moderate	moderate	moderate
Pollock et al. [29]	serious	Serious	low	low	low	moderate	low	serious

* This bias domain can only be assessed to a limited extent since the studies were conducted retrospectively and naturally limited information are available as well as evaluable. ** Prospective observational cohort study—naturally, in an observational study there is no intended intervention.

**Table 5:** ‘Group II—status of sarcoma care’: Limitations and risk bias assessment.

Reference	Study Type	Limitations
Hollunder et al. [30]	Retrospective data analysis *, monocentric	Subjective assessment and missing data as discussed by authors.
Goedhart et al. [31]	Cross-sectional survey	Unvalidated questionnaire as mentioned by authors, non-response bias and selection bias.
Eichler et al. [32]	Observational, prospective cohort study, multicentric	Selection and survivor bias as discussed by authors.
Blank et al. [33]	Retrospective cohort study *, monocentric, prospectively obtained data	Selection bias.The small-sample size and partly retrospectively obtained data are discussed by authors.
Eichler et al. [34]	Cross-sectional survey	Selection-bias and convenience sampling as discussed by authors.
Cho et al. [35]	Cross-sectional survey	Selection bias and small sample-size is discussed by authors. Non-response bias.
Fayet et al. [36]	Retrospective cohort study *, multicentric	For this review relevant results were only of a descriptive nature. Selection bias as discussed by authors.
Weaver et al. [37]	Qualitative interviews	Semi-structured qualitative interview according to COREQ guidelines [46]. Selection bias, non-response bias.
Gantzer et al. [38]	Retrospective data base study *, multicentric, prospectively obtained data	Retrospectively obtained data as discussed by the authors.
George et al. [39]	Retrospective interviews and data analysis *, monocentric	Results only of descriptive nature. The authors discuss a high risk for recall bias due to retrospectively obtained data and selection bias.
Johnson et al. [40]	Retrospective interviews and data analysis *, monocentric	Results only of descriptive nature. The authors discuss a high risk for recall bias due to retrospectively obtained data and selection bias.
Pohlig et al. [41]	Cross-sectional survey	Non-response bias and selection bias.
Styring et al. [42]	Retrospective cohort analysis	Results only of descriptive nature. Selection bias.
Thway et al. [43]	Retrospective analysis *, monocentric	Subjective assessment of endpoint, measurement of outcome
Thway et al. [44]	Retrospective analysis *, monocentric	Subjective assessment of endpoint, measurement of outcome
Wellauer et al. [45]	Retrospective data analysis *	Authors discuss small-sample size and potential subjective assessment of study endpoint.

* Retrospectively obtained data should always be evaluated carefully.

### 2.2. Data Items

For all included studies, the following items were reported: the author, year of publication, study type, the number of patients or participants, and characteristics such as age, sex, and sarcoma type, if applicable. In addition, descriptive data for diagnostics and treatment at an expert centre (EC), respectively, at a non-expert centre (NEC), presentation at an interdisciplinary tumour board (ITB), and compliance to its recommendations were demonstrated. Finally, if valuable for the present review, other results were added in a separate section.

The available data for postoperative outcomes, overall survival (OS), progression-free survival (PFS), disease-specific survival (DSS), disease-free survival (DFS), local recurrence (LR), local recurrence-free survival (LRFS), recurrence-free survival (RFS), metastatic disease (MD), and hazard ratio (HR) retrospectively odds ratio (OR) were extracted. If the topic was not addressed in the study or data were missing, the respective data were not included.

## 3. Results

**Figure 1:**
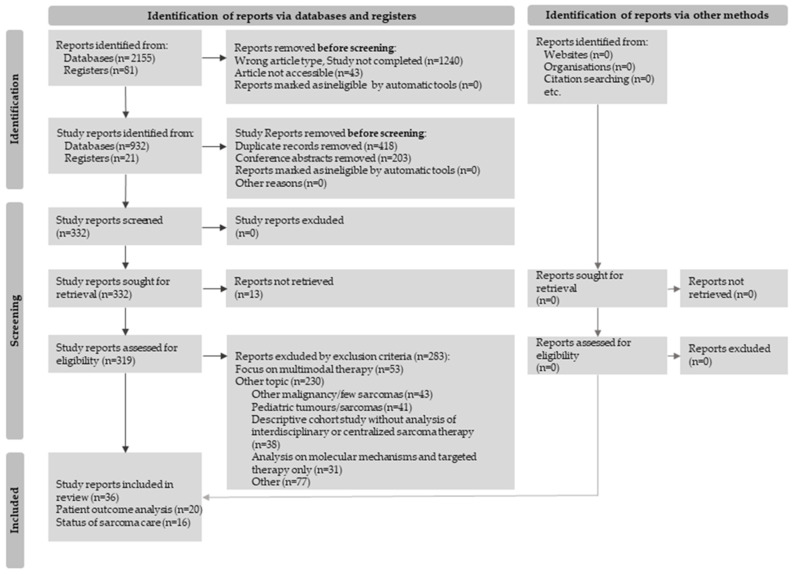
Flow diagram for study selection in accordance with the PRISMA 2020 guidelines [8].

The following PRISMA flow diagram (Figure 1) shows the entire process, from initial identification consisting of all search results to the screening of potential studies until eligible studies are included.

### 3.1. Centralized Treatment at Expert Centres vs. Non-Expert Centres

#### 3.1.1. Access to Specialist Sarcoma Care

Multiple efforts have been made to outline the difficulties in accessing specialised diagnostics or consultation at an expert centre. By interviewing 60 Australian patients, relatives, and health professionals, Weaver et al. identified four main problems in sarcoma care: the patient’s perception and symptoms, difficulties in diagnosing sarcomas, the availability of health services, and lack of experience [37]. Eichler et al. observed similar problems in a cross-sectional survey contacting over two-hundred physicians involved in sarcoma care in Germany. About 30% of participants reported some uncertainty in the treatment of sarcomas, and about one-third reported access problems to diagnostic tools such as FDG-PET and therapy modalities such as deep regional hyperthermia or isolated limb perfusion. Nevertheless, 52.8% of participants from practices cooperated with an expert centre, and 45.7% of participants from level I hospitals were an expert centre itself [34]. In a French cohort study by Fayet et al., 57.9% of the patients in mainland France had access to a specialised expert centre [36]. Another cross-sectional survey by Goedhart et al. with European healthcare professionals reported rates between 5 and 95% of sarcoma patients referred to an expert centre. A total of 20–98% stated that sarcoma patients were treated at expert centres [31]. Through retrospective data analysis and interviews of patients in an expert centre in the United Kingdom, Johnson et al. found a median referral time of 40.4 weeks to an expert centre from the onset of symptoms of sarcoma patients and 25.0 weeks from the first presentation to a health professional [40]. Styring et al. observed a median referral time to an expert centre of 50 days in a Swedish retrospective cohort study [42]. Similar timescales were described by George et al., whose retrospective data analysis and interviews with patients at a British expert-centre found the median delay of referral to an expert centre to be 1 month for soft-tissue sarcomas and 1.5 months for bone sarcomas. Only 4% of soft-tissue sarcomas and 10% of bone sarcomas were referred to an expert centre directly after the initial presentation [39] (Table 3, columns 8–10).

#### 3.1.2. Implementation of Adequate Diagnostic and Pathological Diagnosis

Several data sets are of interest to elucidate the processes involved in suitable diagnostic procedures. In a prospective cohort study at an Australian centre concerning the biopsies of musculoskeletal tumours, Pollock et al. found that adequate material for diagnosis was better obtained when performed by an expert [29]. A cross-sectional survey by Pohlig et al. contacting orthopaedists at tumour centres in Germany showed that 100% of the 25 participating orthopaedic surgeons performed open biopsies, and 72% performed punch biopsies. A proportion of 8% also stated to perform fine-needle biopsies. In addition, 56% of participants reported planning the biopsy access route in an informal meeting and 20% in an interdisciplinary tumour board. Pathologic results were also discussed by 88% in a multidisciplinary tumour board [41]. As a result of a French retrospective data base study of soft-tissue sarcomas by Gantzer et al., adequate imaging and biopsies according to clinical practice guidelines were more often performed at an expert centre. If patients were diagnosed at a non-expert-centre, there was a 3.62-foldand 4.89-fold higher chance for inadequate biopsy and imaging [38]. Bonvalot et al. reported similar results, based on the French NETSARC database, of retroperitoneal soft-tissue sarcomas: patients treated at an expert centre received more frequently adequate imaging and biopsies as well as a presurgical presentation at an interdisciplinary tumour board [17]. In the Swedish retrospective cohort analysis by Styring et al., 86 of 100 included patients were referred to an expert centre before primary surgery, 74 patients received imaging before the referral, and, in 38 cases, a fine needle biopsy was performed [42].

Two consecutive retrospective studies by Thway et al. analysed the discrepancies in the pathological diagnosis of soft-tissue sarcomas and GIST between a British expert and the referring non-expert centre. They found that in 73.4% and 57.8% of cases, the pathological diagnosis of the expert centre was consistent with that of the referring non-expert centre, respectively. However, significant discrepancies between the pathological diagnoses were found in 15.7% and 10.9% of the cases, respectively, resulting in alterations in therapeutic approaches. In the rest of the cases, only minimal or minor discrepancies were observed [43,44]. In a comparable study by Wellauer et al., similar results were found with differences in the pathologic diagnosis between the expert and the referring pathologist. Major discrepancies in pathologic diagnosis leading to a change in treatment management were noted in 12.2% of cases. In addition, expert pathologists established faster diagnoses after receiving the specimen (3.3 days vs. 4.7) [45] (Table 2, column 8; Table 3, column 8).

#### 3.1.3. Overall and Recurrence-Free Survival

It remains an important question whether treatment at specialised centres impacts the prognosis and survival of sarcoma patients. In a retrospective cohort study of the French sarcoma network NETSARC with over 25,000 sarcoma patients by Blay et al., primary surgery at an expert centre was associated with improved overall survival and local recurrence-free survival, stating a hazard ratio of 0.68 and 0.65, respectively [28]. A Portuguese retrospective database study documented similar results for sarcoma patients with a hazard ratio of 0.60 for overall survival if treatment was performed at an expert centre [26]. In Spain, Martin-Broto et al. conducted a prospective, multicentre study of patients with localised soft tissue sarcomas. In line with the previously mentioned studies, treatment at an expert centre was associated with a lower rate of local recurrence and metastasis. The 3-year recurrence-free survival for treatment at an expert centre was 66% compared with 46% for treatment at a non-expert centre [19]. Venigalla et al. made concordant observations based on the National Cancer Database of the United States: patients treated at an expert centre had a better median and 5-year overall survival. The hazard ratio accounted for 0.81 regarding the treatment at an expert centre for overall survival. Bhangu et al. retrospectively studied patients with soft tissue sarcomas treated in the United Kingdom. For therapy in a non-specialised centre, a hazard ratio of 1.7 for overall survival was reported.

Additionally, they observed a lower local relapse rate with treatment at an expert centre [18]. By contrast, in another Spanish retrospective cohort study of patients with localised soft-tissue sarcomas, Abellan et al. observed no effect on overall survival depending on whether patients were referred to their expert centre directly, after primary surgery, or after local recurrence. However, local recurrences were more frequent, and disease-specific survival was poorer when patients were referred to the expert centre after a first local relapse. In addition, metastases were diagnosed more often in this patient group [25].

Several analyses have investigated these effects in retroperitoneal sarcoma. Primary surgery for retroperitoneal soft tissue sarcomas at an expert centre was associated with a better 2-year overall survival and hazard ratio of 0.49 in retrospective analysis based on the French NETSARC database by Bonvalot et al. [17] Similar results were reported by Keung et al. with a retrospective study based on the national cancer database of the United States. Patients with a localized retroperitoneal soft tissue sarcoma profited from a better 5-year overall survival of 57.7% compared to 52% for treatment at an expert centre. Primary surgery at a non-expert-centre was also associated with an increased hazard ratio of 1.8 for overall survival [22]. Snow et al. noted in a retrospective data analysis of an Australian cohort a trend toward reduced risk for local recurrence if the primary surgery of retroperitoneal soft tissue sarcomas was performed at an expert-centre. However, they found that a missing pretherapeutic biopsy and intralesional resection were related to an increased risk of local recurrence with a hazard ratio of 2.8 [15].

Some very rare subtypes have also been evaluated. For example, clear cell sarcomas were analysed in a Polish retrospective cohort study by Ipach et al. The median overall survival was improved, and metastasis occurred later if patients were treated at an expert centre [24]. In PEComas, Sobiborowicz et al. observed a lower local recurrence rate and longer disease-specific survival in a small retrospective cohort study at their centre in Poland [27].

For Ewing sarcomas, Jagodzińska-Mucha et al. observed an improvement in progression-free and 5-year overall survival when patients were referred to a Polish expert centre within three months after the diagnostic biopsy. The hazard ratio for overall survival was 1.6 if this period was longer than three months [11]. Alvarez et al. reported a hazard ratio of 0.49 and 0.78 for overall survival in Ewing sarcoma and osteosarcoma patients, respectively, and a hazard ratio of 0.49 and 0.80 for disease-specific survival if treatment was received at an expert centre [23] (Table 2, column 12–13).

#### 3.1.4. Surgical Outcome

Several data sets are available to assess the influence of surgical outcomes at specialised centres. All of the included studies examining surgical outcomes reported a positive impact on the results when the resection of sarcoma was performed at an expert centre.

Many studies demonstrated that high-volume expert centres more often achieved an R0-resection and lower rates of R1/R2-resections compared to non-expert centres [12,17,20,21,22,24,27,28,38]. Furthermore, focusing on surgical outcomes in soft tissue sarcoma patients, several trials reported a lower rate of positive tumour margins when primary surgery was performed at an expert centre [13,18,19].

In addition, Sandrucci et al. investigated retroperitoneal sarcomas and found that at an expert centre tumours were more often removed intact and that an R0/R1-status was associated with better overall survival [20]. On the other hand, Gilg et al. demonstrated that R1/R2-resections were accompanied by an increased risk for local recurrence in a cohort of myxofibrosarcoma patients [12].

Pollock et al. documented that if an expert had already performed the biopsy, re-resection, and amputations were less likely to be necessary [29]. Additionally, Blay et al. found a lower re-resection rate at expert centres [28]. The results of Keung et al. in a cohort of patients with retroperitoneal soft tissue sarcomas showed a lower 30-day-readmission rate and 30-day-mortality rate after surgery, reflecting lower postoperative complications [22] (Table 2, column 14; Table 3, column 10).

#### 3.1.5. Adjuvant and Neoadjuvant Treatment

Several institutions have investigated perioperative therapy’s influence on localised soft tissue sarcoma. Martin-Broto et al. found that patients with high-risk localised soft tissue sarcomas received perioperative chemotherapy more often at an expert centre. In this prospective Spanish study, perioperative chemotherapy in patients with high-risk constellations was independently associated with a reduced risk of recurrence and improved recurrence-free survival [19]. Ray-Coquard et al. made different observations: chemotherapy in localized soft tissue sarcomas had no significant impact on local recurrence [21]. Soft tissue sarcoma patients included in the retrospective study by Venigalla et al. were more likely to undergo neoadjuvant treatment or chemotherapy if treated at an expert centre. Patients treated at a non-expert centre received more often adjuvant radiotherapy. In comparison, the expert centres treated higher numbers of high-risk patients [13].

Snow et al. reported a higher proportion of neoadjuvant radiotherapy when treated at an expert centre regarding the perioperative treatment of retroperitoneal soft tissue sarcomas. In contrast, adjuvant radiotherapy was used more frequently at non-expert centres. Neoadjuvant radiotherapy was also identified as an independent factor of a lower risk of local recurrence [15]. Bonvalot et al. detected that at their expert centre, an adjuvant treatment was generally more often administered than at a non-expert centre [17]. In the study of patients with retroperitoneal soft tissue sarcoma by Keung et al., chemotherapy was performed with greater frequency at expert centres, and radiotherapy was performed with greater frequency at non-expert centres. Radiotherapy positively affected overall survival with an associated hazard ratio of 0.8 [22]. In treating Ewing sarcomas, Alvarez et al. found an equal rate of radiotherapy when performed at expert and non-expert centres in the United States. However, there was a trend toward a higher rate of patients undergoing chemotherapy at expert centres. Osteosarcoma patients received significantly more chemotherapy at an expert centre [23] (Table 2, column 10, 12–13).

### 3.2. Access and Utilization of an Interdisciplinary Tumour Board

When analysing relevant factors in treating soft tissue sarcoma patients, interdisciplinary tumour boards are of interest. In the Europe-wide survey by Goedhart et al., 47.1% of participants reported access to a multidisciplinary tumour board. A total of 100% of the participating expert centres held a compulsory interdisciplinary tumour board before starting therapy. In 84%, this took place weekly, and an average of 15 cases were discussed [31]. In the German PROsa study by Eichler et al., all participating study centres stated to have the opportunity to participate in an interdisciplinary tumour board, with 44% of them in a tumour board specializing in sarcomas. A total of 88.3% of patients were presented to a tumour board at some point in their therapy, only 56.1% before starting treatment. If treatment was provided at an expert centre, patients had a fivefold higher chance of having their case presented to an interdisciplinary tumour board [32]. In the aforementioned German cross-sectional study also conducted by Eichler et al., 98.7% of participating health professionals at a tertiary hospital and 89% from private practices presented their sarcoma patients to an interdisciplinary tumour board. A total of 93% of participants from tertiary hospitals and 22.2% from private practices stated that they had access to a specialized multidisciplinary sarcoma board [34].

In comparison, in an international survey by Cho et al. to members of the Connective Tissue Oncology Society and the Canadian Association of Radiation Oncology, 96.6% reported having access to a specialised sarcoma board. A total of 85.5% reported presenting every case, and 14.6% only complicated cases [35]. Fayet et al. reported that in France, 30.8% of sarcoma patients diagnosed between 2011 and 2014 had been discussed in a specialised tumour board before primary surgery [36]. In addition, Blay et al. described that an interdisciplinary tumour board led to the increased implementation of adequate tumour imaging and diagnostic biopsy [10]. A retrospective study by Blank et al. carried out at a US cancer centre monitored the performance of national guidelines and showed that in 97% and 94% of cases, the imaging and pathological diagnosis were revised by the multidisciplinary tumour board, respectively. In addition, a treatment plan was discussed for each patient in an interdisciplinary tumour board [33]. Hollunder et al. described that 59.1% of the recommendations of the multidisciplinary tumour board were carried out entirely and 14.4% partially in a retrospective analysis of sarcomas and musculoskeletal tumours at a German Cancer Centre. However, in 8.8% of the cases, these were not implemented for various reasons (e.g., a decision by the treating physician, a patient’s request, or therapy by another institution) [30]. In a retrospective analysis regarding compliance with the tumour board recommendations, Kreyer et al. documented that 77% of the recommendations for Ewing sarcoma were implemented [16] (Table 2, column 9; Table 3, column 8–9).

#### 3.2.1. Overall and Recurrence-Free Survival

In the analysis by Blay et al., an interdisciplinary tumour board before treatment initiation was associated with an increased risk of death with a hazard ratio of 1.56 for overall survival. By contrast, this approach was a favourable prognostic factor for local recurrence-free survival (hazard ratio: 0.67) and disease-free survival (hazard ratio: 0.80) [28]. A more consistent conclusion was also drawn by Blay et al. in another retrospective study based on the NETSARC database of patients with soft tissue sarcomas. If treatment was started before discussions in an interdisciplinary tumour board, this was associated with a higher risk of recurrence. The hazard ratio for local recurrence-free survival was 1.8, and for recurrence-free survival, 1.3 [10]. In the retrospective analysis by Ray-Coquard et al., a lower local recurrence rate was found if patients were presented to a tumour board before surgery [21]. Compliance with the recommendations of the interdisciplinary tumour board in treating Ewing-sarcoma patients was related to an improvement in overall survival. This effect was especially noted in a metastasised stage [16].

By contrast, Marec-Bérard et al. and Bonvalot et al. could not identify the use of an interdisciplinary tumour board as an independent prognostic factor in the treatment of sarcomas [14,17] (Table 2, column 12–13).

#### 3.2.2. Surgical Outcome

Blay et al. observed that fewer re-resections were necessary. An R0 status was often achieved when soft-tissue sarcoma patients were discussed in the interdisciplinary tumour board before starting therapy [10]. Comparable results were presented by Ray-Coquard et al. They showed that patients with localised soft tissue sarcoma presented to an interdisciplinary tumour board more frequently, resulting in an R0 resection than an R2 resection [21]. Kreyer et al. did not find an impact of the recommendation by an interdisciplinary tumour board on the achievement of wide or radical tumour margins in the resection of Ewing-sarcomas [16] (Table 2, column 14).

## 4. Discussion

### 4.1. Synopsis

Bringing together interdisciplinary diagnostics, therapy planning, and treatment strategies at cancer centres have grown to be highly significant for the quality and the outcome of multiple cancer diagnoses, as well as in soft tissue sarcoma. In this systematic review, several significant advantages of the implementation of therapy and treatment at expert centres for soft tissue sarcoma were identified in the present literature.

The included studies and cross-sectional surveys dealing with diagnostic procedures showed that the recommended diagnostic steps were more often enforced at expert centres. This could be shown regarding the correct imaging, obtaining the biopsy material, and integrating a possible pre-therapeutic interdisciplinary tumour board. However, it also revealed that there were uncertainties in sarcoma treatment: not all the necessary resources were always accessible, and diagnosis could be delayed due to missing access to expert treatment.

In almost all studies that conducted a survival time analysis, patients benefited from an improvement in overall or recurrence-free survival when treated at an expert centre. This was reflected in the results of large database analyses on sarcomas in general and up to small cohort studies on specific sarcoma subtypes. Regardless of higher proportions of high-risk patients as described in some studies, a profit for patients treated at expert centres was evident. Furthermore, all studies on surgical outcomes showed higher rates of complete tumour resections when surgery was performed at an expert centre.

According to the guidelines, sarcoma patients should receive multimodal, interdisciplinary therapy [2,4]. In the studies included in this review, neoadjuvant therapy or a multimodal concept was more frequently carried out at expert centres. Supposedly, the rationale for more complex treatment concepts at expert centres was partly explained by more patients presenting at an advanced tumour stage. The application of chemotherapy was repeatedly identified as an adverse prognostic factor. Still, depending on the underlying study, the respective tumour characteristics and risk constellations that led to chemotherapy should be considered.

Regarding the interdisciplinary tumour board, contradictory results were found in the included studies. An advantage of a better surgical outcome in sarcoma patients was described several times if presented in an interdisciplinary tumour board before surgery. The repeated observation of lower local recurrence rates and an improvement in recurrence-free survival was consistent with these results. In one study, however, a presentation in the interdisciplinary tumour board was associated with reduced overall survival. It is probable that in the respective studies, these patients were more likely to be presented to a tumour board due to a higher risk of a constellation.

### 4.2. Limitations

This review aimed to provide an overall view of the current literature. However, the statements made in this review should be taken with caution and consideration of their limitations. A comprehensive database search was conducted using subjectively selected terms, which could not guarantee the comprehensiveness of the topic under review. It should also be noted that differences in the spelling of the term centre might not have been taken into account in some of the database searches. Therefore, it must be considered that the search was not entirely complete. Furthermore, the issue of interdisciplinary therapy is a comparatively vague term, so inclusion or exclusion is also based on a subjective assessment by the reviewer. For the reviewed topic, mainly retrospective studies were found. However, three were conducted prospectively. It should be noted that some databases included in this study are prospective, but they analyse data retrospectively. Regarding the bias assessment, sixteen studies applicable to using the ROBINS-I tool were connected with a severe bias risk, including one critical and only two moderate (Table 4).

The ROBINS-I tool has proven to be effective in comparing non-randomised intervention studies in terms of bias risk with an ideal, randomised, and prospectively conducted the study. However, the ROBINS-I tool has limitations when it comes to the evaluation of retrospective studies: a planned intervention can often not be postulated, which is central to the bias assessment by the ROBINS-I tool [9]. Therefore, especially in the bias domain, “intended intervention bias” can only be assessed to a limited extent. Consequently, it has to be taken into account that in this review, most of the included studies were of a retrospective and observational nature.

The recognition of bias and limitations of the remaining studies for which the ROBINS-I tool was not to be used was, therefore, subject to the individual assessment of the reviewers (Table 5). However, in general, these studies were vulnerable to selection bias.

Concerning the studies presented above, the primary and secondary study endpoints were chosen and examined very differently. However, a well-founded analysis of this approach and its significance for the patient needs clear endpoints and criteria. Therefore, there is a need to clearly define these endpoints to create comparability among individual studies, derive measures for good action, and substantiate them through clear evidence.

Furthermore, a large proportion of the studies focused on surgical outcomes in a localised tumour stage. Patients with metastatic disease seem to be underrepresented concerning centralisation and interdisciplinary therapy. Another topic not addressed in this review is the increasing role of targeted therapy and the implementation of a molecular tumour board.

Several of the included studies were surveys. However, it must be assumed that mainly people with more extensive experience and interest in sarcomas have an intrinsic interest in participating in surveys. Therefore, physicians and institutes that rarely encounter sarcomas may not be adequately represented here.

### 4.3. Centralisation and Interdisciplinary Therapy Management in Other Tumour Entities

In 2014, the European Partnership for Action against cancer (EPAAC) formulated a policy statement that focused on patient-centred therapy. For an optimal, evidence-based implementation of diagnostics and treatment, a multidisciplinary team is crucial to accompany the patient from the very first moment of their disease. In addition to the obligatory presentation on a tumour board, this statement especially emphasises a focus on psychosocial aspects, survival, and comorbidities [47]. Among common tumour entities, the introduction of multidisciplinary therapy and the establishment of expert centres with defined quality standards were also investigated in several studies regarding their influence on patient outcomes.

In a review, Scott et al. summarised the current evidence on different tumour entities and their treatment by a multidisciplinary team: a large proportion of the reviewed studies conducted between 2020 and 2021 showed an improvement in the outcome and overall survival of patients with breast, prostate, lung, colorectal, oesophageal and breast cancer [48]. Berardi et al. summarised the advantages and disadvantages of the tumour board based on an extensive review of the literature: patients benefit from a better outcome, and the interdisciplinary tumour board helps in decision-making and improves cooperation. It also creates better access to expert knowledge, especially in rare diseases with no guidelines. The multidisciplinary tumour board is associated with better adherence if guidelines are available. However, this is limited by the high time expenditure and costs. The quality of the tumour board depends on how information and cases are presented, and tumour boards and national networks are not always accessible. Furthermore, not every included study and tumour entity could observe a significant influence on the outcome [49].

In treating breast cancer patients, a lot of pioneering work has been conducted on multidisciplinary therapy. For example, as early as 2000, the European Society of Breast Cancer Specialists (EUSOMA) published a paper defining the quality standards for breast cancer centres [50].

Kesson et al. published a retrospective cohort study of more than 13,000 women on implementing a multidisciplinary breast cancer care team that served one region in Scotland. This led to an 18% reduction in mortality compared to the surrounding areas. A retrospective study by Tsai et al. examined more than 18,000 breast cancer patients. Treatment by a multidisciplinary team reduced the risk of local recurrence (hazard ratio of 0.84 (95%-CI 0.70–0.99)) [51]. Finally, Roohan et al. compared retrospective registry data from the 1980s of breast cancer patients treated at low-volume and high-volume centres in the state of New York (USA). Patients’ mortality risk was 60% (95%-CI 42–81%) higher if treatment was received at a low-volume centre compared to a high-volume centre. In general, an association of higher case numbers with a lower mortality risk was observed [52].

A Germany-wide cohort of pancreatic cancer patients was retrospectively studied by Roessler et al. Treatment at a centre certified by the German Cancer Society was associated with the near doubling of median overall survival (4.4 vs. 8 months) and a reduced risk of death (hazard ratio 0.89 (95%-CI 0.85–0.93)) [53]. In addition, Brauer et al. prospectively investigated the introduction of an interdisciplinary tumour board in treating patients with pancreatic and upper gastrointestinal tumours. Presentation to a multidisciplinary tumour board revealed a treatment change in 25.1% of the cases. However, an influence on survival could not be observed in the case of pancreatic cancer [54].

A comparable, retrospective study of German-certified cancer centres was conducted by Völkl et al. on patients with localised colorectal carcinoma. In an accredited centre, the 3-year survival rate was 71.6% compared to 63.3% in non-certified centres. Overall, reduced risk with a hazard ratio of (HR = 0.808, 95%-CI 0.665–0.982) was found with treatments at a certified “expert centre” [55]. Peng et al. undertook a meta-analysis that included 11 trials and 30,000 patients with colorectal cancer. Regardless of tumour stage, patients treated by a multidisciplinary team benefited from an improvement in overall survival (HR 0.81, 95%-CI 0.69–0.94). By contrast, no effect on postoperative mortality was found [56].

### 4.4. Achievements in Interdisciplinary Sarcoma Treatment

The therapeutic principles investigated in the studies mentioned above also play a significant role in treating rare cancers. For example, European health professionals, patient organisations, and industry members designed the Sarcoma Policy Checklist as a joint venture. In this, the development of national sarcoma centres as centres of high-quality sarcoma treatment and the need for multidisciplinary teams was emphasised as essential principles to optimise sarcoma treatment [57].

A positive example of implementing these principles into sarcoma therapy is the French clinical reference network for soft tissue and visceral sarcomas NETSARC. This implies a comprehensive network of centres, experts, and guideline-based diagnostics and therapy enforcement. Every patient is obligatorily presented in an interdisciplinary sarcoma board and is automatically included in the database registry. Furthermore, the histology of each patient is reviewed by a reference pathologist [10,58]. Blay et al. recently reported on the success associated with the establishment of NETSARC: throughout France, improved overall survival was observed for both localised and metastasised sarcomas [Hazard ratio 0.82; Hazard ratio 0.68]. In addition, there was an improvement in the overall course of sarcoma treatment: a higher proportion was biopsied before starting therapy as well as presented to an interdisciplinary tumour board. Furthermore, a growing proportion of patients were also operated on at one reference centre [59].

About 20 years ago, the German Cancer Society’s certification program for cancer centres began to take shape. This program focused on multidisciplinary therapies that followed the highest quality standards and the current guidelines. In 2019, 56% of all cancer patients in Germany were treated at certified cancer centres [60]. In April 2022, it was reported that treatment at a centre certified by the German Cancer Society reduced the mortality risk of cancer patients by up to 26% [60,61]. The certification of sarcoma centres by the German Cancer Society was introduced four years ago. Currently, nineteen centres have been certified according to the criteria of the German Cancer Society [62].

### 4.5. Conclusions

The studies examined in this review show a high degree of heterogeneity, and their conclusive statements are partly discrepant. However, there is growing evidence that patients benefit from treatment at expert centres and the integration of an interdisciplinary tumour board as a meeting point for all those involved in sarcoma therapy. It appears that these established structures improve the outcomes of sarcoma patients through multidisciplinary action.

There have been multiple efforts in establishing these structures toward better networking and the centralisation of treating sarcoma patients to provide access to a standardised, guideline-based therapy. The crucial determinants of multidisciplinary therapy and the development of expert centres for high-quality treatment, among others, have been defined by national and international guidelines as well as reports, such as the Sarcoma Policy Checklist or the Rare Cancer Agenda 2030, but also by national programs, such as the certification program of the German Cancer Society or the NETSARC network in France [2,4,6,7,28,63]. The task is to implement these structures, as we see the first successes for sarcoma therapy, for example, in France [59].

Broad databases will help to unambiguously identify quality indicators, a lack of resources, and where to integrate them into an up-to-date guideline-based therapy. Furthermore, this will increase awareness of interdisciplinary management and cooperation with centres of expertise in sarcoma treatment, aiming to eliminate all treatment uncertainties outside and inside expert centres in the future.

## Figures and Tables

**Table 1:** Inclusion and exclusion criteria.

Category	Inclusion Criteria	Exclusion Criteria
Article type	-	Guidelines, reviews, expert opinions, editorials, letters
Study design	Any except reviews/systematic reviews/case series Retrospective/prospective/observational/intervention study	Systematic review Case reports/series < 10 patients
Population	Age: focus on patients > 18 years Diagnosis of sarcoma according to WHO-classification Histologic grade: any Stage: any	Exclusively paediatric and adolescent patients Diagnosis other than sarcoma
Investigation	Diagnostic/Treatment at expert centre (EC) Interdisciplinary tumour board (ITB) presentation Status quo of access to EC/ITB	Multimodal therapy Pharmaceutical study
Outcome	Postoperative outcome Disease-specific survival (DSS) disease-free survival (DFS) Local recurrence (LR), Local recurrence-free survival, metastatic disease (MD) Overall survival (OS) Progression-free survival (PFS) Recurrence-free survival (RFS)	
Date range	Until 12 July 2022	

## Data Availability

Not applicable.

## Supplementary Materials

The following supporting information can be downloaded at: https://www.mdpi.com/article/10.3390/life13040979/s1, File S1: PRISMA2020-Checklist.
